# Cross‐Sectional and Longitudinal Associations of Irisin and Adiponectin With Obesity, Sarcopenia and Sarcopenic Obesity

**DOI:** 10.1002/jcsm.70172

**Published:** 2025-12-29

**Authors:** Yejin Kim, Hong Ji Song, Dong‐Hyun Kim, Jin‐Young Jeong, Kyung Hee Park, Yong Soon Park, Jwa‐Kyung Kim, Hye‐Mi Noh

**Affiliations:** ^1^ Department of Medical Sciences, College of Medicine Hallym University Chuncheon Republic of Korea; ^2^ Department of Family Medicine, Hallym University Sacred Heart Hospital, College of Medicine Hallym University Anyang Republic of Korea; ^3^ Department of Social and Preventive Medicine, Hallym University College of Medicine Chuncheon South Korea; ^4^ Hallym Research Institute of Clinical Epidemiology, Hallym University College of Medicine Chuncheon South Korea; ^5^ Department of Family Medicine, Chuncheon Sacred Heart Hospital, College of Medicine Hallym University Chuncheon South Korea; ^6^ Department of Internal Medicine & Kidney Research Institute, Hallym University Sacred Heart Hospital, College of Medicine Hallym University Anyang Republic of Korea

**Keywords:** adiponectin, aging, irisin, obesity, sarcopenia, sarcopenic obesity

## Abstract

**Background:**

Irisin, an exercise‐induced myokine and adiponectin, an adipocyte‐derived hormone, are involved in energy metabolism and musculoskeletal health. However, their associations with obesity, abdominal obesity, sarcopenia and sarcopenic obesity remain unclear, and longitudinal evidence is limited. This study investigated the cross‐sectional and longitudinal associations of these biomarkers with obesity‐ and sarcopenia‐related outcomes.

**Methods:**

We used data from the Hallym Aging Study, a cohort of Korean adults aged ≥ 45 years (45–64 years: 30%, ≥65 years: 70%). The cross‐sectional analysis used the third wave (2010), while the longitudinal analysis included the second (2007, baseline) and third waves. In both analyses, sex‐specific tertile groups (T1, T2 and T3) were defined based on baseline levels and changes in irisin and adiponectin over 3 years. Lean soft tissue and fat mass were assessed using dual‐energy x‐ray absorptiometry. Obesity was defined as body fat percentages > 29.7% for males and > 37.2% for females. For individuals without obesity, low ALST was defined as appendicular lean soft tissue (ALST)/height^2^ < 7.0 kg/m^2^ for males and < 5.4 kg/m^2^ for females. For individuals with obesity, low ALST was defined as an ALST/body weight < 26.8% for males and < 21.0% for females. Multivariable logistic regression was performed to examine the associations between circulating irisin and adiponectin and obesity‐ and sarcopenia‐related outcomes.

**Results:**

A total of 357 and 360 participants were included in the cross‐sectional analyses of irisin and adiponectin, respectively, and 351 were included in the longitudinal analysis (baseline 2007; median age: 70 years; 54% female). In cross‐sectional analysis, irisin was not significantly associated with obesity‐ and sarcopenia‐related outcomes after adjustment for confounding variables. Longitudinally, the greatest irisin increase group (T3) over 3 years had higher odds of obesity [odds ratio (OR) 2.39, 95% confidence interval (CI) 1.24–4.71], abdominal obesity (OR 2.19, 95% CI 1.04–4.72), sarcopenia (OR 2.11, 95% CI 1.14–3.97), sarcopenic obesity (OR 3.40, 95% CI 1.43–8.61) and low ALST (OR 2.21, 95% CI 1.24–3.99) at the follow‐up than the T1 group. In contrast, adiponectin levels showed inverse associations with obesity (OR 0.47, 95% CI 0.24–0.93) and abdominal obesity (OR 0.48, 95% CI 0.25–0.90) only in cross‐sectional analysis.

**Conclusions:**

Despite irisin's proposed protective role against obesity and sarcopenia, our findings suggest that greater 3‐year increases in irisin were associated with increased odds of obesity, abdominal obesity, sarcopenia and sarcopenic obesity at the follow‐up. Conversely, adiponectin showed an inverse cross‐sectional association with obesity and abdominal obesity.

## 1 Introduction

The global proportion of older adults is expected to double within three decades, reaching 16% by 2050 [1]. As the global population ages, metabolic dysfunction and physical decline associated with aging have become major public health concerns. Aging is associated with progressive loss of skeletal muscle mass and strength, while many older adults simultaneously experience increased fat accumulation and redistribution, particularly in visceral areas [2]. Sarcopenia, characterized by the loss of muscle mass and function, significantly impacts the quality of life and independence in older adults [3]. Similarly, obesity in the aging population is a significant risk factor for disability, falls, fractures, cardiovascular disease and mortality [4]. The interplay between aging, obesity and sarcopenia has led to the emergence of sarcopenic obesity, a condition characterized by the coexistence of sarcopenia and obesity, which has been associated with more severe metabolic abnormalities and functional limitations than either condition alone [5, 6].

Exercise‐induced signalling molecules called exerkines, secreted by muscle, hear and adipose tissue, are essential for cardiovascular health, metabolic regulation and immune function [7]. Irisin, a myokine generated through the cleavage of fibronectin type III domain‐containing protein 5 (FNDC5), is involved in exercise‐induced metabolic processes. By activating PPAR‐γ coactivator‐1α (PGC‐1α) and uncoupling protein 1 (UCP1), irisin promotes the browning of white adipose tissue, thereby increasing energy expenditure and fat oxidation [8]. However, most human studies have shown a positive relationship between circulating irisin or FNDC5 mRNA abundance and body mass index (BMI) [9, 10, 11]. In addition, higher circulating irisin levels have been observed in individuals with overweight or obesity, suggesting a compensatory response to metabolic dysfunction [12].

Adiponectin, an adipokine secreted by adipose tissue, is well recognized for its metabolic benefits, including increased fatty acid oxidation, anti‐inflammatory effects and improved insulin sensitivity [13]. Additionally, adiponectin contributes to skeletal muscle energy homeostasis and metabolic regulation by modulating mitochondrial function [14]. Exercise has been shown to increase adiponectin levels, which may contribute to improved muscle metabolism, regeneration and reduced inflammation in older adults and aging mice models [15, 16]. However, paradoxically, higher adiponectin levels in older adults have also been associated with lower muscle mass and strength, as well as increased risk of cardiovascular disease and mortality, a phenomenon known as the ‘adiponectin paradox’ [17, 18].

Despite growing interest, the roles of irisin and adiponectin in musculoskeletal health remain unclear. Regarding sarcopenia, some studies have reported lower circulating irisin levels in individuals with sarcopenia and positive associations with muscle mass and strength [9, 19, 20], whereas others found no significant associations [21, 22]. Moreover, the association between adiponectin and sarcopenia remains controversial in older adults. While adiponectin has demonstrated beneficial metabolic effects and has been shown to promote muscle regeneration in experimental models [15], clinical studies have reported paradoxical findings, with both increased and decreased levels observed in individuals with sarcopenia [16, 23, 24].

However, previous studies have investigated these biomarkers in relation to either obesity or sarcopenia independently, without considering the complex interplay between these conditions. Given that sarcopenic obesity represents a distinct clinical phenotype characterized by the coexistence of excess fat and reduced muscle mass and function, it is crucial to assess how irisin and adiponectin are associated with obesity, sarcopenia and sarcopenic obesity simultaneously. Moreover, most studies have been limited to cross‐sectional designs, providing little evidence on how changes in irisin and adiponectin influence these conditions over time. To address these gaps, this study aimed (1) to examine the cross‐sectional associations of circulating irisin and adiponectin with obesity, sarcopenia and sarcopenic obesity in a population‐based cohort of middle‐aged and older Korean adults and (2) to assess how changes in these biomarkers over a 3‐year period are associated with these outcomes at follow‐up.

## 2 Methods

### 2.1 Study Population

The Hallym Aging Study (HAS), initiated in 2003, is a population‐based cohort study investigating the quality of life among adults aged 45 and older in Chuncheon, South Korea. The study design has been described elsewhere [25, 26]. Briefly, Chuncheon was divided into 1408 census‐based areas, from which 200 were randomly selected. Through systematic sampling, 1520 participants were recruited (30% aged 45–64 years; 70% aged ≥ 65 years). The HAS cohort was first assessed in 2004 (*n* = 918), followed by the second wave in 2007 (*n* = 547) and the third wave in 2010 (*n* = 382). For the current analysis, we used data from the 2007 (baseline) and 2010 waves, excluding individuals with missing irisin at both waves.

All study procedures were conducted in accordance with the ethical standards of the 1964 Declaration of Helsinki and its later amendments and were approved by the Institutional Review Board of Hallym University (IRB number: 2024‐09‐001) Written informed consent was obtained from all participants prior to study enrolment. For participants with impaired decision‐making capacity, legally authorized representatives were permitted to provide consent on their behalf.

### 2.2 Assessment of Sarcopenia Components

Appendicular lean soft tissue (ALST), body fat mass and percentage body fat were assessed in 2010 using dual‐energy x‐ray absorptiometry (DXA; Lunar, GE, Fairfield, CT, USA). Given the limitations of BMI in accurately reflecting adiposity and individual health risk [27], obesity was defined based on percentage body fat, with thresholds of > 29.7% for males and > 37.2% for females [28]. Depending on the obesity status, we defined low ALST differently. As ALST adjusted for height squared (ALST/height^2^) may underestimate sarcopenia in individuals with obesity, low ALST in individuals with obesity was defined as ALST/body weight (%), with thresholds of < 26.8% for males and < 21.0% for females [29]. For individuals without obesity, low ALST was defined as ALST/height^2^ < 7.0 kg/m^2^ for males and < 5.4 kg/m^2^ for females according to the 2019 Asian Working Group for Sarcopenia (AWGS) guidelines [30]. Muscular strength was assessed using a digital hand dynamometer (TKK‐5401; Takei, Japan). Handgrip strength was measured twice for each hand, and the highest value was used for analysis. Low muscle strength was defined as handgrip strength < 28 kg for males and < 18 kg for females [30]. Physical performance was evaluated using gait speed and the five‐time sit‐to‐stand test (5‐time STS). For gait speed assessment, participants were instructed to walk at their usual pace over a 6‐m course. For the 5‐time STS test, participants were instructed to cross their arms over their chest and stand up from a chair and sit down five consecutive times. Low physical performance was defined as a gait speed < 1.0 m/s or a 5‐time STS test time ≥ 12 s [30].

### 2.3 Definition of Sarcopenia and Sarcopenic Obesity

Sarcopenia was diagnosed according to the AWGS 2019 definition as the presence of low ALST along with either low muscle strength or low physical performance [30]. Severe sarcopenia was defined as the concurrent presence of low ALST, low muscle strength and low physical performance. Sarcopenic obesity was defined as the presence of both sarcopenia and obesity [31]. The detailed diagnostic algorithm is illustrated in Figure S1.

### 2.4 Questionnaire and Anthropometric Assessments

Structured interviews were conducted to assess sociodemographic and health behaviour factors. Information was collected on age, sex, smoking status, alcohol consumption and exercise habits. Current smoking was defined as having smoked ≥ 400 cigarettes throughout the lifetime and currently smoking, while alcohol drinking was defined as consuming alcohol at least once over the past year. Regular exercise was defined as doing moderate‐intensity exercise for at least 150 min per week. Anthropometric measurements were performed in light clothing without shoes. Height and weight were measured to the nearest 0.1 cm and 0.1 kg, respectively. BMI was calculated as weight divided by height squared (kg/m^2^). Waist circumference (WC) was measured at the midpoint between the lowest rib and iliac crest to the nearest 0.1 cm. Abdominal obesity was defined as WC ≥ 90 cm for males and ≥ 85 cm for females, based on the Korean Society for the Study of Obesity Guidelines [32].

### 2.5 Biochemical Measurements

Blood samples were collected after overnight fasting. Plasma glucose was measured using an auto‐analyser (Hitachi 747, Japan). Plasma insulin concentrations were measured using a gamma counter (Cobra II, Packard Bioscience, Meriden, CT, USA). Insulin resistance was evaluated using homeostasis model assessment of insulin resistance (HOMA‐IR), calculated as [fasting insulin (μU/mL) × fasting glucose (mg/dL)]/405. C‐reactive protein (CRP) was analysed using a particle‐enhanced nephelometric immunoassay (Hitachi 7600 Analyzer; Hitachi Ltd., Japan). Plasma adiponectin levels were assessed using an immunoassay platform (VersaMax, Molecular Devices, Sunnyvale, CA, USA). Plasma irisin was measured using a commercial enzyme‐linked immunosorbent assay (ELISA) kit (NBP3‐08117; Novus Biologicals, Centennial, CO, USA). Samples from 2007 and 2010 were centrifuged, stored at −70°C and thawed prior to analysis. Plasma was diluted 1:20 before measurement, and values outside the assay detection range (0.16–10 ng/mL) were treated as missing data.

### 2.6 Statistical Analysis

Continuous variables were presented as mean ± standard deviation (SD) or median (interquartile range [IQR]) and categorical variables as numbers (percentages). Group differences across irisin and adiponectin tertiles were performed using one‐way analysis of variance (ANOVA) or Kruskal–Wallis tests for continuous variables, and chi‐squared tests for categorical variables. Post hoc analyses were conducted using Tukey's HSD for ANOVA and Dunn's test with Bonferroni correction for Kruskal–Wallis tests. For cross‐sectional analysis, multivariable logistic regression was conducted to examine the associations between sex‐specific tertiles of irisin and adiponectin and obesity‐ and sarcopenia‐related outcomes. Sex‐specific tertiles were applied because of known sex differences [26]. The following three models were applied: Model 1 was unadjusted; Model 2 included demographic variables (age and sex) and health‐related factors (current smoking, alcohol drinking, regular exercise and comorbidity); and Model 3 was further adjusted for metabolic markers (HOMA‐IR and CRP) and mutual adjustment for adiponectin and irisin. Comorbidity was based on self‐reported presence of stroke, myocardial infarction, arthritis, depression, osteoporosis, malignancy, diabetes, dyslipidaemia and hypertension and categorized as none, one or two or more. In the longitudinal analysis, changes in irisin and adiponectin were calculated as the difference between the third (2010) and second (2007) waves, and sex‐specific tertiles were applied. Logistic regression models were used with progressive adjustment. Model 1 was unadjusted. Model 2 was adjusted for baseline biomarker levels and sarcopenia‐related parameters. Because ALST was available only in the third wave (2010), BMI was used as a proxy for baseline adjustments in obesity and sarcopenia‐related outcomes (WC was used for abdominal obesity). Additionally, sarcopenia‐related variables were further adjusted for muscle strength and physical performance. Model 3 included all covariates from Model 2, with additional adjustments for demographic, health‐related and metabolic factors, as well as mutual adjustment for adiponectin and irisin, consistent with cross‐sectional analysis. Additionally, we estimated the odds of obesity‐ and sarcopenia‐related outcomes per 1‐unit increase in log‐transformed irisin and adiponectin levels for the cross‐sectional analysis, and per 1‐SD change in these biomarkers for the longitudinal analysis. Causal mediation analysis was performed to evaluate whether the associations of irisin or adiponectin (independent variable) with obesity, abdominal obesity and sarcopenic obesity (dependent variable) were mediated by HOMA‐IR and CRP. Because of right‐skewed distributions, irisin, adiponectin, HOMA‐IR and CRP were log‐transformed prior to analysis. Linear and logistic regression models were used for mediation analysis, with nonparametric bootstrapping (5000 resamples) applied to estimate 95% confidence intervals (CIs). Mediation effects were considered significant if the 95% CI excluded zero. All models were adjusted for demographic, health‐related and metabolic factors. All analyses were performed using R (Version 4.2.0; R Foundation for Statistical Computing, Vienna, Austria), with statistical significance set at *p* < 0.05.

## 3 Results

**FIGURE 1:**
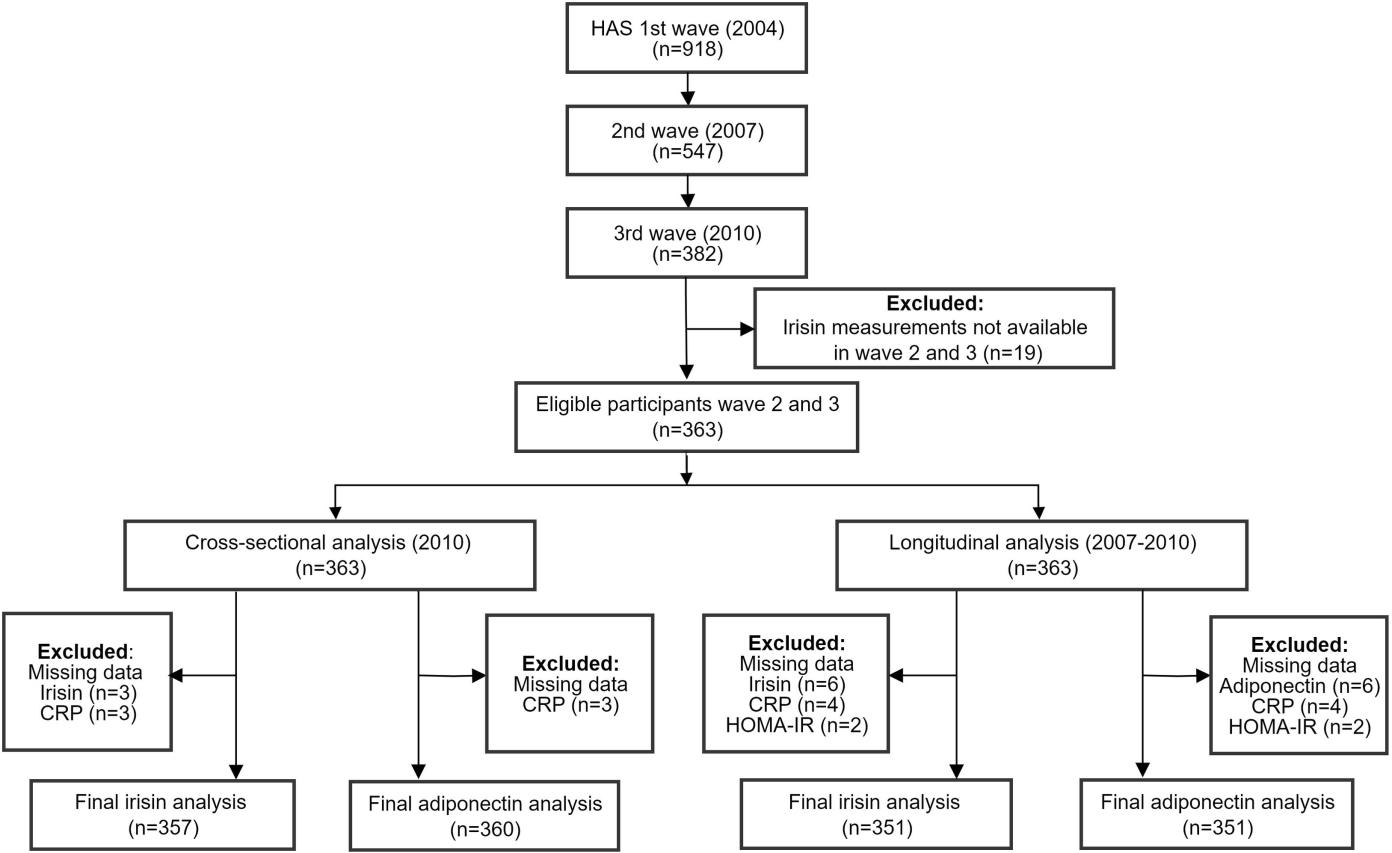
Flow chart of the study participants.

The participant enrolment flowchart is presented in Figure 1. This study utilized data from the 2007 and 2010 waves of the HAS longitudinal cohort. Cross‐sectional analysis used 2010 data when DXA measurements were available. Longitudinal analysis employed 2007 as baseline and 2010 as follow‐up. Of 382 participants who completed both the second and third waves, 19 with missing irisin data at both time points were excluded, yielding 363 participants. Additionally, participants with missing data on irisin, adiponectin, CRP or HOMA‐IR were excluded from analysis.

Participant characteristics by irisin and adiponectin tertiles (*n* = 357 and 360; 54% females; median age 73–75 years) are presented in Table 1. Participants in the highest irisin tertile had a higher BMI (*p* = 0.018), WC (*p* = 0.007), body fat mass (*p* = 0.004), HOMA‐IR (*p* < 0.001) and a higher prevalence of abdominal obesity (*p* = 0.009) and sarcopenic obesity (*p* = 0.046) compared to the lowest tertile. In contrast, the highest adiponectin tertile presented older age (*p* = 0.001); lower HOMA‐IR, BMI, WC, body fat mass and percentage body fat (all *p* < 0.001); and ALST/height^2^ (*p* = 0.023) than the lowest tertile group. The prevalence of obesity, abdominal obesity (both *p* < 0.001) and sarcopenic obesity (*p* = 0.015) was significantly lower in the highest adiponectin tertile group.

**TABLE 1:** Characteristics of participants by irisin and adiponectin tertiles (2010).

	Irisin (*n* = 357)				Adiponectin (*n* = 360)			
Variable	Tertile 1 (*n* = 120)	Tertile 2 (*n* = 119)	Tertile 3 (*n* = 118)	*p* ^a^	Tertile 1 (*n* = 121)	Tertile 2 (*n* = 120)	Tertile 3 (*n* = 119)	*p* ^a^
Range of irisin (ng/mL)/adiponectin level (μg/mL)	Male: <57.7 Female: <38.4	Male: 57.7–105.0 Female: 38.4–62.0	Male: >105.0 Female: >62.0		Male: <6.2 Female: <8.4	Male: 6.2–9.7 Female: 8.4–12.8	Male: >9.7 Female: >12.8	
**Baseline characteristics**								
Age (years)	73.0 (69.5:76.0)	74.0 (69.0:77.0)	74.0 (71.0:77.0)	0.84	73.0 (69.0:75.0)^d^	73.0 (68.0:78.0)^d^	75.0 (72.0:79.0)^b^, ^c^	**0.001**
Female	65 (54.2%)	65 (54.6%)	64 (54.2%)	0.997	66 (54.5%)	65 (54.2%)	65 (54.6%)	0.997
Current smoking	6 (5.0%)	17 (14.3%)	12 (10.2%)	0.054	15 (12.4%)	12 (10.0%)	9 (7.6%)	0.459
Alcohol drinking	28 (23.3%)	37 (31.1%)	44 (37.3%)	0.064	39 (32.2%)	31 (25.8%)	39 (32.8%)	0.429
Regular exercise	30 (25.0%)	36 (30.3%)	31 (26.3%)	0.636	37 (30.6%)	29 (24.2%)	31 (26.1%)	0.514
Comorbidity				0.273				0.463
No comorbidity	21 (17.5%)	29 (24.4%)	20 (16.9%)		23 (19.0%)	23 (19.2%)	24 (20.2%)	
1 condition	43 (35.8%)	33 (27.7%)	32 (27.1%)		33 (27.3%)	33 (27.5%)	43 (36.1%)	
2 or more conditions	56 (46.7%)	57 (47.9%)	66 (55.9%)		65 (53.7%)	64 (53.3%)	52 (43.7%)	
**Body composition**								
Body mass index (kg/m^2^)	24.0 (22.6:26.5)^d^	24.3 (22.1:26.2)^d^	25.6 (23.4:27.5)^b^, ^c^	**0.018**	25.7 (23.9:27.6)^d^	25.1 (22.7:27.0)^d^	23.1 (21.3:24.9)^b^, ^c^	**<0.001**
Waist circumference (cm)	84.9 ± 9.7^d^	84.9 ± 8.3^d^	88.1 ± 8.6^b^, ^c^	**0.007**	88.7 ± 8.1^d^	86.9 ± 8.0^d^	81.9 ± 9.6^b^, ^c^	**<0.001**
Abdominal obesity	51 (42.5%)	45 (38.1%)	67 (57.3%)	**0.009**	66 (54.5%)	65 (54.6%)	32 (27.1%)	**<0.001**
Body fat mass (kg)	17.8 ± 8.1^d^	19.0 ± 7.3	21.3 ± 8.0^b^	**0.004**	21.7 ± 7.1^d^	20.3 ± 8.4^d^	16.6 ± 7.7^b^, ^c^	**<0.001**
Percentage body fat (%)	29.1 ± 9.8	30.1 ± 9.2	31.0 ± 8.9	0.29	31.6 (25.1:38.2)^d^	31.4 (24.8:39.1)^d^	27.5 (20.0:34.4)^b^, ^c^	**<0.001**
Obesity	28 (23.3%)	40 (33.6%)	41 (34.7%)	0.108	45 (37.2%)	44 (36.7%)	20 (16.8%)	**<0.001**
**Sarcopenic components**								
ALST/height^2^ (kg/m^2^)	6.19 (5.56:7.18)	6.06 (5.60:6.86)	6.22 (5.53:7.19)	0.522	6.47 (5.84:7.19)^d^	6.13 (5.62:7.04)	5.97 (5.46:6.88)^b^	**0.023**
ALST/weight (%)	26.4 (22.5;29.3)	25.5 (22.5;28.8)	25.6 (21.2;28.5)	0.317	25.5 (22.2;28.5)	25.1 (21.7;29.0)	26.7 (22.5;29.5)	0.079
Low ALST	44 (36.7%)	55 (46.2%)	57 (48.3%)	0.154	47 (38.8%)	59 (49.2%)	51 (42.9%)	0.266
Grip strength (kg)	24.1 (19.4:33.2)	22.8 (19.4:32.8)	25.8 (18.8:33.0)	0.677	24.8 (19.6:34.5)	23.6 (19.7:32.6)	23.6 (18.3:32.8)	0.213
Low muscle strength	27 (22.5%)	35 (29.4%)	28 (23.7%)	0.423	26 (21.5%)	26 (21.7%)	39 (32.8%)	0.071
Gait speed (m/s)	1.00 (0.87:1.19)	1.03 (0.85:1.16)	0.99 (0.78:1.18)	0.724	1.05 (0.88:1.16)	1.00 (0.85:1.20)	0.99 (0.78:1.18)	0.402
Chair 5‐time STS (s)	12.3 (10.0:15.8)	11.8 (9.9:15.2)	12.5 (10.2:14.8)	0.912	12.1 (9.6:15.5)	12.3 (9.9:14.6)	12.6 (10.5:16.5)	0.389
Low physical performance	85 (70.8%)	72 (60.5%)	80 (67.8%)	0.222	77 (63.6%)	80 (66.7%)	82 (68.9%)	0.686
Sarcopenia	31 (25.8%)	38 (31.9%)	44 (37.3%)	0.164	36 (29.8%)	37 (30.8%)	40 (33.6%)	0.802
Sarcopenic obesity	11 (9.2%)	16 (13.4%)	24 (20.3%)	**0.046**	23 (19.0%)	20 (16.7%)	8 (6.7%)	**0.015**
Severe sarcopenia	30 (25.0%)	36 (30.3%)	43 (36.4%)	0.159	12 (9.9%)	14 (11.7%)	12 (10.1%)	0.888
**Biochemical variables**								
Irisin (ng/mL)	29.7 (19.1:37.6)^c^, ^d^	58.8 (47.9:77.8)^b^, ^d^	114.8 (86.0:150.6)^b^, ^c^	**< 0.001**	62.0 (37.6:107.9)	55.7 (38.2:91.9)	57.4 (36.2:86.9)	0.392
Adiponectin (μg/mL)	9.35 (6.60:14.30)	9.20 (6.75:12.55)	8.70 (5.90:12.60)	0.238	5.50 (4.20:6.60)^c^, ^d^	9.10 (8.20:10.1)^b^, ^d^	15.6 (13.4:20.4)^b^, ^c^	**<0.001**
HOMA‐IR	1.13 (0.59:1.93)^d^	1.15 (0.66:1.92)^d^	1.58 (0.97:2.69)^b^, ^c^	**<0.001**	1.68 (1.10–2.98)^c^, ^d^	1.22 (0.76–2.00)^b^, ^d^	0.97 (0.47–1.50)^b^, ^c^	**<0.001**
CRP (mg/dL)	0.08 (0.05:0.19)	0.12 (0.06:0.20)	0.10 (0.06:0.20)	0.241	0.12 (0.06:0.20)	0.10 (0.06:0.20)	0.08 (0.05:0.19)	0.116

*Note:* Continuous variables are presented as mean ± SD for normal distributions and as median (IQR) for nonnormal distributions. Categorical variables are expressed as *n* (%).

^a^ Statistical analyses used ANOVA for normal distributions, Kruskal–Wallis for nonnormal distributions and chi‐squared tests for categorical variables. Post hoc analyses were conducted using Tukey's test for ANOVA and Dunn's test with Bonferroni correction for Kruskal–Wallis.

^b^ *p* < 0.05 versus Tertile 1;

^c^ *p* < 0.05 versus Tertile 2;

^d^ *p* < 0.05 versus Tertile 3. *p* values < 0.05 are presented in bold.

Abbreviations: ALST, appendicular lean soft tissue; Chair 5‐time STS, Chair 5‐time sit‐to‐stand; CRP, C‐reactive protein.

**FIGURE 2:**
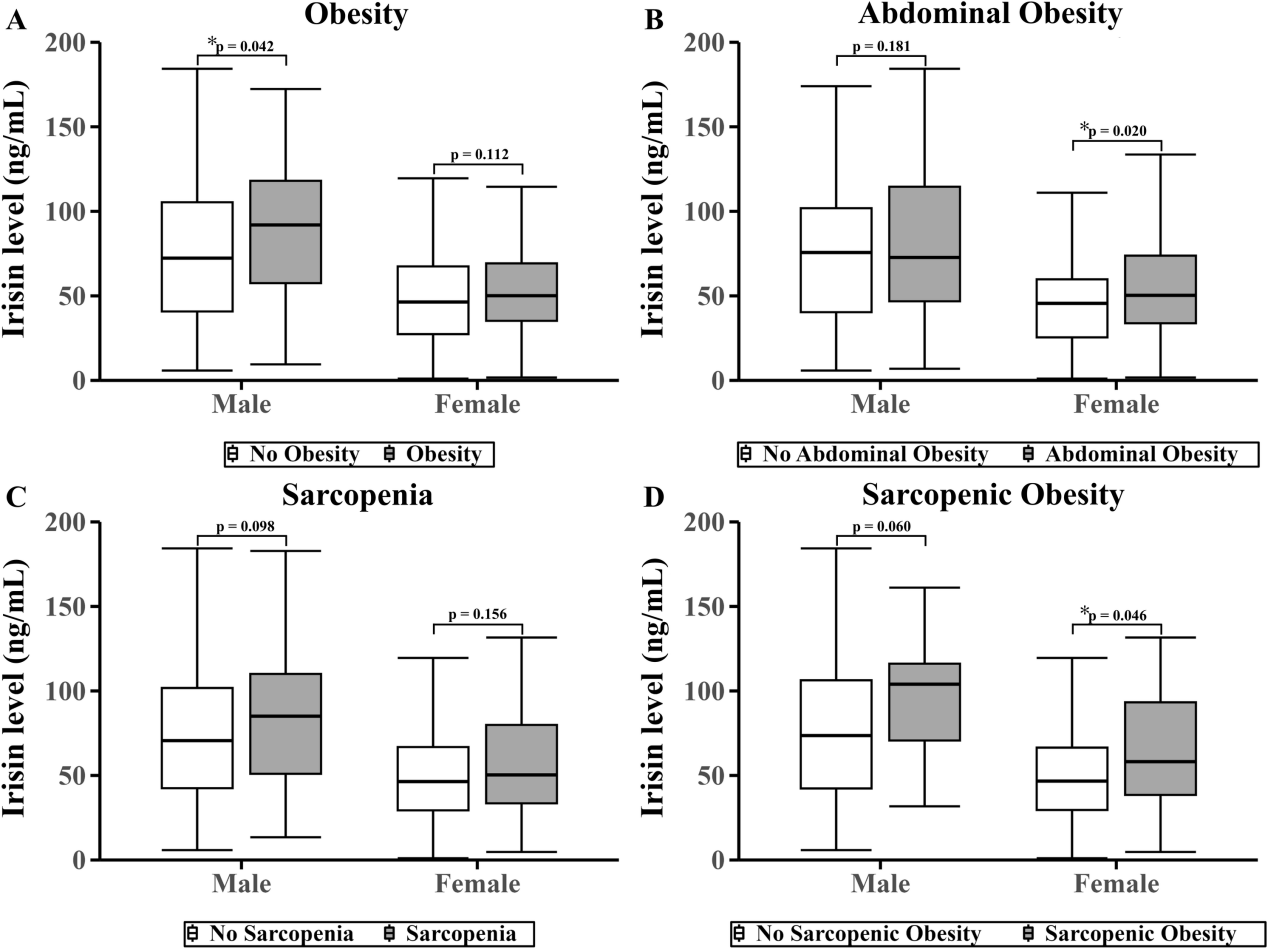
Circulating irisin levels in male and female by (A) obesity, (B) abdominal obesity, (C) sarcopenia and (D) sarcopenic Obesity status. Box plots show irisin levels in participants with and without each condition. Outliers beyond 1.5 times the IQR were excluded from the plots. Statistical comparisons were conducted using the Mann–Whitney *U* test, and *p* values are shown above each comparison. ^✻^ *p* < 0.05; ^✻✻^ *p* < 0.01.

**FIGURE 3:**
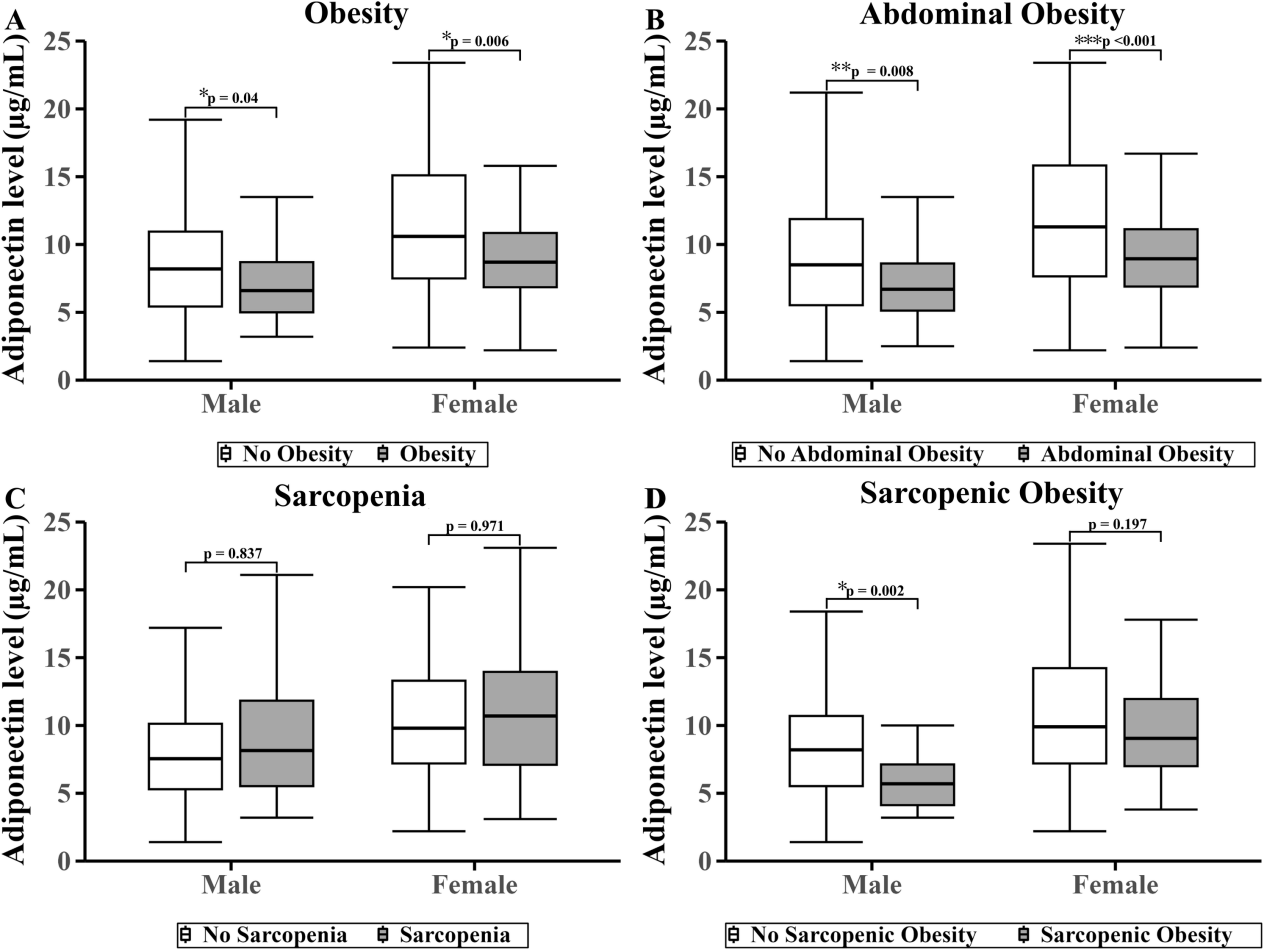
Circulating adiponectin levels in male and female by (A) obesity, (B) abdominal obesity, (C) sarcopenia and (D) sarcopenic Obesity status. Box plots show adiponectin levels in participants with and without each condition. Outliers beyond 1.5 times the IQR were excluded from the plots. Statistical comparisons were conducted using the Mann–Whitney *U* test, and *p* values are shown above each comparison. ^✻^ *p* < 0.05; ^✻✻^ *p* < 0.01; ^✻✻✻^ *p* < 0.001.

Differences in irisin levels according to obesity, abdominal obesity, sarcopenia and sarcopenic obesity are presented separately by sex in Figures 2 and 3. In males, irisin levels were significantly higher in those with obesity than without (101.6 vs. 76.7 ng/mL; *p* = 0.042). In females, irisin levels were significantly higher in those with abdominal obesity (52.7 vs. 45.7 ng/mL; *p* = 0.020) and sarcopenic obesity (60.0 vs. 47.6 ng/mL; *p* = 0.046). For adiponectin, males showed significantly lower levels in those with obesity (6.70 vs. 8.20 μg/mL; *p* = 0.040), abdominal obesity (6.70 vs. 8.70 μg/mL; *p* = 0.008) and sarcopenic obesity (5.70 vs. 8.30 μg/mL; *p* = 0.002). In females, adiponectin levels were significantly lower in those with obesity (9.05 vs. 11.00 μg/mL; *p* = 0.006) and abdominal obesity (9.2 vs. 12.6 μg/mL; *p* < 0.001).

Table 2 shows the cross‐sectional associations between irisin and adiponectin tertiles and obesity‐ and sarcopenia‐related outcomes. Participants in the highest irisin tertile had significantly higher odds of obesity (odds ratio [OR] 1.85, 95% CI 1.03–3.35), abdominal obesity (OR 1.82, 95% CI 1.06–3.14) and sarcopenic obesity (OR 2.59, 95% CI 1.18–5.97) compared to the lowest tertile in Model 2. However, these associations lost significance after further adjustment for HOMA‐IR, CRP and adiponectin. For adiponectin, participants in the highest tertile had significantly lower odds of obesity (OR 0.47, 95% CI 0.24–0.93) and abdominal obesity (OR 0.48, 95% CI 0.25–0.90) compared to the lowest tertile in Model 3. When analysed as continuous variables (per 1‐unit increase in log‐transformed irisin and adiponectin levels; Table S1), irisin was not associated with obesity‐ and sarcopenia‐related outcomes. Adiponectin was inversely associated with obesity, abdominal obesity and sarcopenic obesity, but these associations were no longer significant after further adjustment for HOMA‐IR, CRP and irisin.

**TABLE 2:** Cross‐sectional associations of irisin and adiponectin tertiles with obesity‐ and sarcopenia‐related outcomes (2010).

	Irisin (*n* = 357)					Adiponectin (*n* = 360)				
	T1	T2, OR (95% CI)	*p*	T3, OR (95% CI)	*p*	T1	T2, OR (95% CI)	*p*	T3, OR (95% CI)	*p*
**Obesity**										
Model 1	1	1.66 (0.95–2.96)	0.079	1.75 (1.00–3.11)	0.054	1	0.98 (0.58–1.65)	0.933	0.34 (0.18–0.62)	**0.001**
Model 2	1	1.78 (0.99–3.23)	0.056	1.85 (1.03–3.35)	**0.041**	1	0.98 (0.57–1.68)	0.94	0.35 (0.18–0.65)	**0.001**
Model 3	1	1.72 (0.93–3.21)	0.083	1.45 (0.78–2.72)	0.237	1	1.32 (0.74–2.38)	0.343	0.47 (0.24–0.93)	**0.031**
**Abdominal obesity**										
Model 1	1	0.83 (0.50–1.40)	0.493	1.81 (1.09–3.05)	**0.024**	1	0.94 (0.56–1.56)	0.805	0.30 (0.17–0.51)	**<0.001**
Model 2	1	0.87 (0.50–1.50)	0.606	1.82 (1.06–3.14)	**0.031**	1	0.92 (0.54–1.56)	0.748	0.29 (0.16–0.51)	**<0.001**
Model 3	1	0.78 (0.43–1.41)	0.409	1.35 (0.75–2.45)	0.315	1	1.50 (0.83–2.71)	0.182	0.48 (0.25–0.90)	**0.022**
**Sarcopenia**										
Model 1	1	1.35 (0.77–2.37)	0.299	1.71 (0.98–2.99)	0.058	1	1.05 (0.61–1.83)	0.855	1.20 (0.69–2.07)	0.52
Model 2	1	1.35 (0.75–2.45)	0.317	1.68 (0.94–3.03)	0.08	1	0.98 (0.55–1.74)	0.945	1.16 (0.65–2.08)	0.611
Model 3	1	1.29 (0.71–2.35)	0.405	1.54 (0.85–2.80)	0.155	1	1.14 (0.63–2.07)	0.674	1.39 (0.76–2.58)	0.287
**Severe sarcopenia**										
Model 1	1	0.85 (0.37–1.92)	0.695	0.86 (0.37–1.94)	0.711	1	1.20 (0.53–2.75)	0.662	1.02 (0.43–2.39)	0.966
Model 2	1	0.90 (0.36–2.20)	0.818	0.82 (0.33–2.01)	0.664	1	0.87 (0.35–2.13)	0.756	0.65 (0.25–1.65)	0.367
Model 3	1	0.88 (0.35–2.18)	0.781	0.78 (0.31–1.94)	0.586	1	0.93 (0.37–2.33)	0.877	0.69 (0.26–1.80)	0.448
**Sarcopenic obesity**										
Model 1	1	1.54 (0.69–3.56)	0.299	2.53 (1.20–5.63)	**0.017**	1	0.85 (0.44–1.65)	0.635	0.31 (0.12–0.69)	**0.006**
Model 2	1	1.53 (0.66–3.66)	0.326	2.59 (1.18–5.97)	**0.02**	1	0.82 (0.41–1.63)	0.564	0.32 (0.13–0.76)	**0.013**
Model 3	1	1.19 (0.49–2.97)	0.701	1.84 (0.81–4.39)	0.153	1	1.13 (0.53–2.42)	0.748	0.43 (0.15–1.11)	0.09
**Low ALST**										
Model 1	1	1.48 (0.89–2.50)	0.135	1.61 (0.96–2.72)	0.07	1	1.52 (0.91–2.55)	0.107	1.18 (0.71–1.98)	0.527
Model 2	1	1.50 (0.87–2.61)	0.144	1.62 (0.94–2.80)	0.085	1	1.56 (0.92–2.68)	0.103	1.27 (0.73–2.21)	0.392
Model 3	1	1.46 (0.85–2.55)	0.174	1.51 (0.87–2.64)	0.143	1	1.78 (1.02–3.12)	**0.043**	1.43 (0.81–2.57)	0.221
**Low muscle strength**										
Model 1	1	1.44 (0.80–2.58)	0.224	1.07 (0.59–1.96)	0.822	1	1.01 (0.55–1.87)	0.973	1.78 (1.00–3.20)	0.05
Model 2	1	1.50 (0.79–2.89)	0.219	1.03 (0.53–2.01)	0.935	1	0.83 (0.42–1.62)	0.585	1.39 (0.74–2.65)	0.31
Model 3	1	1.48 (0.78–2.86)	0.234	0.98 (0.50–1.94)	0.963	1	0.91 (0.45–1.80)	0.778	1.44 (0.74–2.81)	0.283
**Low physical performance**										
Model 1	1	0.63 (0.37–1.08)	0.094	0.87 (0.50–1.50)	0.612	1	1.14 (0.67–1.95)	0.622	1.27 (0.74–2.17)	0.388
Model 2	1	0.56 (0.30–1.02)	0.06	0.84 (0.45–1.57)	0.582	1	1.04 (0.58–1.89)	0.888	1.06 (0.57–1.96)	0.851
Model 3	1	0.55 (0.29–1.00)	0.051	0.80 (0.42–1.52)	0.501	1	1.14 (0.62–2.10)	0.667	1.20 (0.64–2.27)	0.575

*Note:* Model 1: unadjusted; Model 2: adjusted for age, sex, current smoking, alcohol drinking, regular exercise and comorbidity; Model 3: Model 2 + HOMA‐IR, CRP and mutual biomarkers (adiponectin and irisin, respectively). Each biomarker tertile was divided into sex‐specific tertiles. For irisin (ng/mL), in male, T1, <57.7; T2, 57.7–105.0; T3, >105.0; in female, T1, <38.4; T2, 38.4–62.0; T3, >62.0. For adiponectin (μg/mL), in male, T1, <6.2; T2, 6.2–9.7; T3, >9.7; in female, T1, <8.4; T2, 8.4–12.8; T3, >12.8. *p* values < 0.05 are presented in bold.

Abbreviations: ALST, appendicular lean soft tissue; CI, confidence interval; OR, odds ratio; T1; Tertile 1, T2, Tertile 2; T3, Tertile 3.

Table 3 presents baseline, change and follow‐up characteristics by sex‐specific tertiles of irisin and adiponectin changes. At follow‐up, participants with the greatest increase in irisin (Tertile 3) had greater increases in WC (*p* = 0.003), a higher prevalence of obesity (*p* = 0.016) and abdominal obesity (*p* = 0.014), higher body fat mass (*p* = 0.021) and faster gait speed (*p* = 0.014) compared to those with the least increased or decreased irisin (Tertile 1). For adiponectin, those with the greatest increase (Tertile 3) had significantly greater decreases in BMI (*p* < 0.001) and WC (*p* < 0.001).

**TABLE 3:** Baseline (2007), changes and follow‐up (2010) measures by irisin and adiponectin change tertiles.

	Irisin (*n* = 351)				Adiponectin (*n* = 351)			
Variable	Tertile 1 (*n* = 117) Least increased or decreased	Tertile 2 (*n* = 117) Intermediate change	Tertile 3 (*n* = 117) Greatest increase	*p* ^a^	Tertile 1 (*n* = 118) Greatest decrease	Tertile 2 (*n* = 117) Intermediate change	Tertile 3 (*n* = 116) Greatest increase	*p* ^a^
**Range of irisin change (ng/mL)/adiponectin change (μg/mL)**	Male: <10.6 Female: <2.5	Male: 10.7–30.0 Female: 2.6–19.4	Male: >30.0 Female: >19.4		Male: <−1.7 Female: <−2.7	Male: −1.6–0.6 Female: −2.6–0.5	Male: >0.6 Female: >0.5	
Irisin change (2007–2010)	−8.24 (−25.5:‐0.88)^c,d^	15.3 (11.0:19.8)^b^, ^d^	42.8 (31.6:59.4)^b^, ^c^	**<0.001**	17.5 (1.36:31.6)	15.4 (−3.01:37.6)	12.9 (−2.67:26.4)	0.207
Adiponectin change (2007–2010)	−0.60 (−2.70:1.40)	−1.10 (−3.70:1.65)	−1.00 (−3.50:0.75)	0.407	−4.60 (−6.80:‐3.20)^c^, ^d^	−0.80 (−1.30:‐0.20)^b^, ^d^	2.25 (1.35:4.35)^b^, ^c^	**<0.001**
**Baseline characteristics**								
Age (years) (2007)	71.0 (67.0:74.0)	70.0 (66.0:73.0)	70.0 (65.0:74.0)	0.364	70.0 (66.0:73.0)	70.0 (67.0:74.0)	72.0 (66.0:74.5)	0.295
Female	64 (54.7%)	64 (54.7%)	64 (54.7%)	1	64 (54.2%)	64 (54.7%)	63 (54.3%)	0.997
Current smoking (2007)	8 (6.8%)	12 (10.3%)	19 (16.2%)	0.068	15 (12.7%)	11 (9.4%)	15 (12.9%)	0.642
Alcohol drinking (2007)	39 (33.3%)	25 (21.4%)	39 (33.3%)	0.068	25 (21.2%)	37 (31.6%)	40 (34.5%)	0.062
Regular exercise (2007)	32 (27.4%)	34 (29.1%)	32 (27.4%)	0.945	37 (31.4%)	26 (22.2%)	35 (30.2%)	0.238
Comorbidity (2007)				0.503				0.491
No comorbidity	32 (27.4%)	31 (26.5%)	34 (29.1%)		34 (28.8%)	29 (24.8%)	33 (28.5%)	
1 condition	42 (35.9%)	33 (28.2%)	42 (35.9%)		45 (38.1%)	39 (33.3%)	34 (29.3%)	
2 or more conditions	43 (36.8%)	53 (45.3%)	41 (35.0%)		39 (33.1%)	49 (41.9%)	49 (42.2%)	
**BMI (kg/m** ^**2**^)								
BMI (2007)	24.6 (22.0:26.3)	24.3 (22.7:26.0)	25.3 (23.2:27.2)	0.132	24.7 (22.4:26.3)	24.3 (22.8:26.8)	24.5 (22.5:26.7)	0.832
BMI change (2007–2010)	0.20 (−0.90:1.10)	0.20 (−0.70:0.90)	0.00 (−0.60:1.00)	0.87	0.63 (−0.20:1.40)^c^, ^d^	−0.10 (−0.80:0.70)^b^	−0.30 (−1.25:0.90)^b^	**<0.001**
**WC (cm)**								
WC (2007)	85.6 ± 9.5	84.6 ± 8.4	85.9 ± 7.4	0.443	84.4 ± 8.7	85.8 ± 8.3	85.8 ± 8.3	0.351
WC change (2007–2010)	0.30 (−4.50:3.00)^d^	0.65 (−3.85:3.40)^d^	2.00 (−1.00:5.00)^b^, ^c^	**0.003**	2.50 (−1.00:5.50)^c^, ^d^	0.60 (−3.70:3.60)^b^	−0.20 (−4.80:2.00)^b^	**<0.001**
Abdominal obesity (2007)	52 (44.4%)	42 (35.9%)	52 (44.4%)	0.31	43 (36.4%)	54 (46.2%)	48 (41.4%)	0.319
Abdominal obesity (2010)	50 (43.1%)	44 (37.9%)	66 (56.4%)	**0.014**	57 (48.7%)	51 (43.6%)	49 (42.6%)	0.603
**Body fat (2010)** ^e^								
Body Fat Mass (kg)	18.5 ± 8.0^d^	19.4 ± 8.0	21.3 ± 7.6^b^	**0.021**	19.9 ± 7.4	20.1 ± 8.2	18.7 ± 8.5	0.353
Percentage Body Fat (%)	29.2 (21.5:35.6)	30.5 (22.8:37.1)	32.3 (25.6:37.8)	0.082	29.5 (23.9:37.7)	31.6 (23.6:38.1)	28.9 (22.0:35.0)	0.149
**Obesity (2010)** ^e^								
Obesity	25 (21.4%)	38 (32.5%)	45 (38.5%)	**0.016**	38 (32.2%)	43 (36.8%)	26 (22.4%)	0.052
**Sarcopenic components (2010)** ^e^								
ALST/height^2^ (kg/m^2^)	6.06 (5.61:7.05)	6.17 (5.58:7.08)	6.29 (5.47:7.02)	0.898	6.04 (5.58:7.19)	6.40 (5.63:6.83)	6.12 (5.45:7.04)	0.754
ALST/weight (%)	26.3 (23.0;28.8)	25.8 (22.3;29.3)	24.6 (21.6;28.4)	0.152	25.9 (22.2;29.0)	25.5 (22.1;28.7)	25.8 (22.3;29.4)	0.727
Low ALST	47 (40.2%)	45 (38.5%)	61 (52.1%)	0.071	44 (37.3%)	59 (50.4%)	51 (44.0%)	0.127
Grip strength (kg)	23.2 (18.7:31.4)	23.9 (19.4:33.8)	24.8 (20.3:32.8)	0.292	24.2 (18.8:33.8)	24.5 (19.9:32.6)	24.2 (19.0:33.2)	0.974
Low muscle strength	33 (28.2%)	26 (22.2%)	28 (23.9%)	0.551	31 (26.3%)	26 (22.2%)	31 (26.7%)	0.682
Walking speed (m/s)	0.97 (0.73:1.11)^c^	1.08 (0.88:1.22)^b^	1.03 (0.86:1.23)	**0.014**	1.00 (0.86:1.20)	1.05 (0.87:1.18)	0.97 (0.76:1.16)	0.127
Chair 5‐time STS (s)	13.2 (10.1:16.7)	12.0 (9.8:14.6)	12.0 (10.2:14.0)	0.233	11.7 (9.6:14.9)	12.3 (9.7:15.3)	12.7 (10.4:16.3)	0.363
Low physical performance	84 (71.8%)	73 (62.4%)	74 (63.2%)	0.245	44 (37.3%)	39 (33.3%)	35 (30.2%)	0.513
Sarcopenia	37 (31.6%)	29 (24.8%)	44 (37.6%)	0.107	32 (27.1%)	38 (32.5%)	41 (35.3%)	0.389
Sarcopenic obesity	12 (10.3%)	14 (12.0%)	24 (20.5%)	0.055	16 (13.6%)	21 (17.9%)	13 (11.2%)	0.327
Severe sarcopenia	14 (12.0%)	9 (7.7%)	14 (12.0%)	0.470	14 (11.9%)	10 (8.5%)	13 (11.2%)	0.681
**Biochemical variables**								
Irisin (2007)	51.7 (32.3:83.2)^c^	36.1 (21.8:58.8)^b^	42.2 (27.4:74.5)	**0.007**	39.9 (26.1:61.4)	46.6 (23.5:76.9)	49.7 (30.2:83.2)	0.07
Adiponectin (2007)	9.00 (6.50:13.7)	11.50 (7.55:15.0)	9.90 (7.05:14.3)	0.137	13.6 (10.2:19.0)^c^, ^d^	8.30 (6.10:11.2)^b^	8.70 (5.95:12.6)^b^	**<0.001**
**HOMA‐IR**								
HOMA‐IR (2007)	1.86 (1.17:3.14)	1.76 (1.43:2.93)	2.14 (1.49:3.62)	0.123	2.10 (1.37:3.59)^d^	2.10 (1.52:3.31)^d^	1.63 (1.15:2.50)^b^, ^c^	**0.007**
HOMA‐IR change (2007–2010)	−0.59 (−1.53:0.07)	−0.58 (−1.43:0.14)	−0.52 (−1.49:0.17)	0.948	−0.67 (−1.64:0.15)	−0.63 (−1.45:‐0.02)	−0.39 (−1.39:0.15)	0.407
**CRP (mg/dL)**								
CRP (2007)	0.09 (0.05:0.25)	0.09 (0.05:0.16)	0.09 (0.06:0.18)	0.429	0.09 (0.05:0.18)	0.10 (0.06:0.21)	0.08 (0.05:0.18)	0.289
CRP change (2007–2010)	0.00 (−0.05:0.05)	0.01 (−0.02:0.05)	0.00 (−0.04:0.06)	0.316	0.00 (−0.03:0.06)	−0.00 (−0.04:0.04)	0.01 (−0.04:0.09)	0.417

*Note:* Continuous variables are presented as mean ± SD for normal distributions and as median (IQR) for nonnormal distributions. Categorical variables are expressed as *n* (%). Baseline and follow‐up measurements were used to calculate change variables as the difference between values from the third (2010) and the second (2007) waves

^a^ Statistical analyses used ANOVA for normal distributions, Kruskal–Wallis for nonnormal distributions and chi‐squared tests for categorical variables. Post hoc analyses were conducted using Tukey's test for ANOVA and Dunn's test with Bonferroni correction for Kruskal–Wallis.

^b^ *p* < 0.05 versus Tertile 1;

^c^ *p* < 0.05 versus Tertile 2;

^d^ *p* < 0.05 versus Tertile 3. *p* values < 0.05 are presented in bold.

^e^ Variables assessed by DXA and sarcopenic components represent the value in 2010, while all other variables not named as ‘change’ refer to 2007 measurements

Abbreviations: ALST, appendicular lean soft tissue; BMI, body mass index; Chair 5‐time STS, Chair 5‐time sit‐to‐stand; CRP, C‐reactive protein; WC, waist circumference.

Table 4 presents the longitudinal associations of 3‐year changes in irisin and adiponectin with sarcopenia‐related parameters at follow‐up. Those with the greatest increase in irisin (Tertile 3) had significantly higher odds of obesity (OR 2.39, 95% CI 1.24–4.71), abdominal obesity (OR 2.19, 95% CI 1.04–4.72), sarcopenia (OR 2.11, 95% CI 1.14–3.97), sarcopenic obesity (OR 3.40, 95% CI 1.43–8.61) and low ALST (OR 2.21, 95% CI 1.24–3.99) compared to Tertile 1 after full adjustment. For adiponectin, those with the greatest increase (Tertile 3) showed a trend toward lower odds of obesity (OR 0.52, 95% CI 0.25–1.06), abdominal obesity (OR 0.46, 95% CI 0.20–1.02) and severe sarcopenia (OR 0.40, 95% CI 0.14–1.15) compared to Tertile 1; however, these associations were not statistically significant. In contrast, participants with intermediate changes (Tertile 2) showed significantly lower odds of abdominal obesity (OR 0.34, 95% CI 0.14–0.80), but higher odds of low ALST (OR 1.90, 95% CI 1.03–3.53) compared with Tertile 1.

**TABLE 4:** Longitudinal associations of changes in irisin and adiponectin with obesity‐ and sarcopenia‐related outcomes at follow‐up.

	Irisin (*n* = 351)					Adiponectin (*n* = 351)				
	T1	T2, OR (95% CI)	*p*	T3, OR (95% CI)	*p*	T1	T2, OR (95% CI)	*p*	T3, OR (95% CI)	*p*
**Obesity**										
Model 1	1	1.77 (0.99–3.21)	0.057	2.30 (1.30–4.14)	**0.005**	1	1.22 (0.71–2.10)	0.463	0.61 (0.34–1.08)	0.094
Model 2	1	2.11 (1.10–4.16)	**0.027**	2.32 (1.22–4.47)	**0.011**	1	1.22 (0.64–2.36)	0.549	0.53 (0.26–1.05)	0.069
Model 3	1	2.05 (1.04–4.14)	**0.04**	2.39 (1.24–4.71)	**0.01**	1	1.17 (0.59–2.32)	0.653	0.52 (0.25–1.06)	0.074
**Abdominal obesity**										
Model 1	1	0.81 (0.48–1.36)	0.423	1.71 (1.02–2.88)	0.043	1	0.81 (0.49–1.36)	0.432	0.78 (0.46–1.31)	0.351
Model 2	1	0.87 (0.43–1.75)	0.692	2.11 (1.08–4.19)	0.031	1	0.62 (0.30–1.26)	0.185	0.54 (0.26–1.08)	0.086
Model 3	1	0.86 (0.40–1.88)	0.713	2.19 (1.04–4.72)	0.042	1	0.34 (0.14–0.80)	**0.015**	0.46 (0.20–1.02)	0.06
**Sarcopenia**										
Model 1	1	0.71 (0.40–1.26)	0.246	1.30 (0.76–2.24)	0.337	1	1.29 (0.74–2.27)	0.369	1.47 (0.84–2.57)	0.175
Model 2	1	0.92 (0.50–1.68)	0.783	1.80 (1.01–3.25)	**0.049**	1	1.14 (0.60–2.15)	0.688	1.09 (0.58–2.03)	0.794
Model 3	1	0.81 (0.42–1.54)	0.514	2.11 (1.14–3.97)	**0.019**	1	1.23 (0.63–2.41)	0.548	1.27 (0.65–2.48)	0.483
**Severe sarcopenia**										
Model 1	1	0.61 (0.25–1.46)	0.276	1.00 (0.45–2.22)	1	1	0.69 (0.29–1.62)	0.403	0.94 (0.42–2.10)	0.875
Model 2	1	0.82 (0.31–2.14)	0.693	1.91 (0.78–4.81)	0.16	1	0.45 (0.16–1.22)	0.121	0.44 (0.16–1.16)	0.102
Model 3	1	0.77 (0.25–2.30)	0.637	2.04 (0.73–5.95)	0.181	1	0.34 (0.11–1.03)	0.06	0.40 (0.14–1.15)	0.093
**Sarcopenic obesity**										
Model 1	1	1.19 (0.52–2.73)	0.678	2.26 (1.09–4.91)	**0.033**	1	1.39 (0.69–2.87)	0.357	0.80 (0.36–1.76)	0.586
Model 2	1	1.62 (0.67–4.05)	0.289	2.89 (1.28–6.89)	**0.013**	1	0.99 (0.43–2.32)	0.987	0.48 (0.19–1.20)	0.119
Model 3	1	1.53 (0.59–4.04)	0.383	3.40 (1.43–8.61)	**0.007**	1	1.08 (0.43–2.70)	0.87	0.49 (0.18–1.31)	0.16
**Low ALST**										
Model 1	1	0.93 (0.55–1.57)	0.789	1.62 (0.97–2.73)	0.067	1	1.71 (1.02–2.89)	**0.043**	1.32 (0.78–2.23)	0.299
Model 2	1	1.08 (0.62–1.86)	0.789	1.85 (1.08–3.22)	**0.027**	1	1.62 (0.91–2.90)	0.104	1.17 (0.65–2.09)	0.597
Model 3	1	1.02 (0.57–1.83)	0.954	2.21 (1.24–3.99)	**0.008**	1	1.90 (1.03–3.53)	**0.042**	1.41 (0.76–2.62)	0.278
**Low muscle strength**										
Model 1	1	0.73 (0.40–1.31)	0.293	0.80 (0.44–1.44)	0.457	1	0.80 (0.44–1.46)	0.469	1.02 (0.57–1.83)	0.937
Model 2	1	0.87 (0.44–1.72)	0.682	1.19 (0.60–2.36)	0.618	1	0.65 (0.31–1.37)	0.261	0.89 (0.44–1.82)	0.756
Model 3	1	0.87 (0.40–1.88)	0.716	1.31 (0.61–2.82)	0.494	1	0.55 (0.23–1.28)	0.174	0.77 (0.34–1.72)	0.523
**Low physical performance**										
Model 1	1	0.65 (0.37–1.13)	0.127	0.68 (0.39–1.17)	0.164	1	1.19 (0.70–2.04)	0.526	1.38 (0.80–2.38)	0.25
Model 2	1	0.97 (0.50–1.90)	0.937	1.22 (0.63–2.38)	0.56	1	1.42 (0.70–2.90)	0.336	0.94 (0.46–1.92)	0.867
Model 3	1	0.86 (0.42–1.78)	0.691	1.31 (0.63–2.73)	0.469	1	1.10 (0.51–2.36)	0.811	0.87 (0.40–1.88)	0.727

*Note:* Model 1: unadjusted. Model 2: adjusted for baseline irisin or adiponectin levels, BMI (WC for abdominal obesity) and sarcopenia‐related variables. Specifically, sarcopenia, sarcopenic obesity and low ALST were adjusted for baseline handgrip strength, chair stand test and gait speed. Low muscle strength was adjusted for baseline handgrip strength only, and low physical performance for baseline chair stand test and gait speed. Model 3: Model 2 adjustments plus age, sex, current smoking, alcohol drinking, regular exercise, comorbidity, HOMA‐IR, CRP and mutual biomarkers (adiponectin and irisin, respectively). Each biomarker change was divided into sex‐specific tertiles. For irisin change (ng/mL), in male, T1, <10.6; T2, 10.7–30.0; T3, >30.0; in female, T1, <2.5; T2, 2.6–19.4; T3, >19.4. For adiponectin change (μg/mL), in male, T1, <−1.7; T2, −1.6–0.6; T3, >0.6; in female, T1, <−2.7; T2, −2.6–0.5; T3, >0.5. *p* values < 0.05 are presented in bold.

Abbreviations: CI, confidence interval; OR, odds ratio; T1; Tertile 1, T2, Tertile 2; T3, Tertile 3.

A 1‐SD increase in change of irisin level was associated with higher odds of abdominal obesity (OR 1.42, 95% CI 1.02–2.02) and sarcopenic obesity (OR 1.46, 95% CI 1.03–2.11). In contrast, a 1‐SD increase in change of adiponectin level showed a significant inverse association with abdominal obesity (OR 0.68, 95% CI 0.48–0.95) and severe sarcopenia (OR 0.55, 95% CI 0.31–0.93) (Table S2)

**FIGURE 4:**
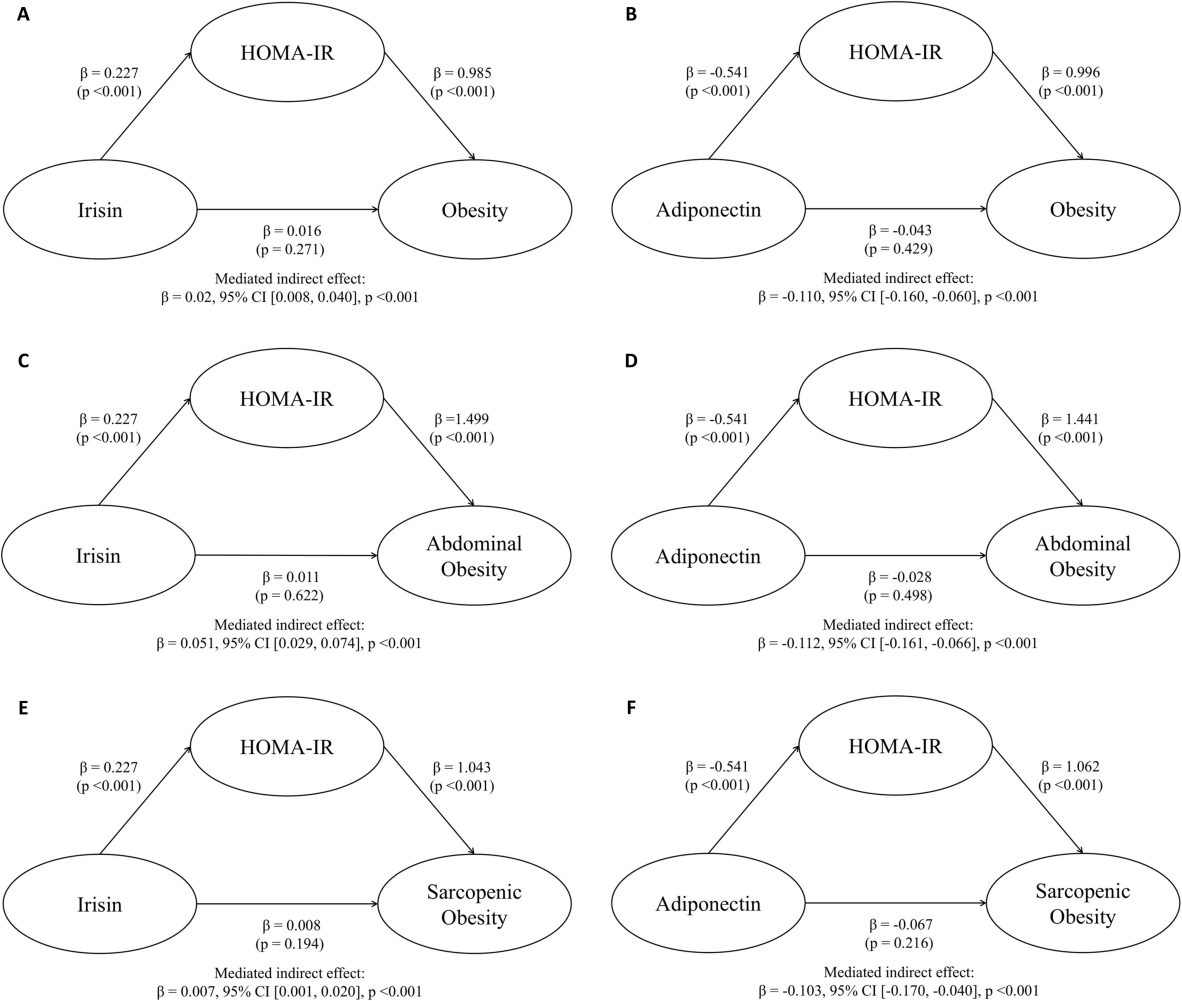
Mediation effects of insulin resistance (HOMA‐IR) on the associations between irisin or adiponectin and obesity (A, B), abdominal obesity (C, D) and sarcopenic obesity (E, F). Analyses were adjusted for age, sex, current smoking, alcohol drinking, regular exercise, comorbidity and CRP. Irisin, adiponectin, HOMA‐IR and CRP were log‐transformed. The 95% confidence intervals were estimated using the bootstrap method (5000 samples).

The mediation analysis showed that the association between irisin, obesity, abdominal obesity and sarcopenic obesity was mediated by HOMA‐IR levels (Figure 4). For irisin, the indirect effects via HOMA‐IR were significant for obesity (β = 0.020, 95% CI [0.008, 0.040]), abdominal obesity (β = 0.051, 95% CI [0.029, 0.074]) and sarcopenic obesity (β = 0.007, 95% CI [0.001, 0.020]), while the direct effects of irisin were not significant. Similarly, for adiponectin, HOMA‐IR showed significant indirect effects on obesity (β = −0.110, 95% CI [−0.160, −0.060]), abdominal obesity (β = −0.112, 95% CI [−0.161, −0.066]) and sarcopenic obesity (β = −0.103, 95% CI [−0.170, −0.040]), while the direct effects of adiponectin on these outcomes were not significant. In contrast, there were no significant indirect effects in the associations between irisin or adiponectin and obesity, abdominal obesity or sarcopenic obesity when CRP was examined as a mediator (Figure S2).

## 4 Discussion

In this population‐based cohort study, we examined the associations of circulating irisin and adiponectin levels with obesity, abdominal obesity, sarcopenia and sarcopenic obesity in both cross‐sectional and longitudinal analyses. In the cross‐sectional analysis, higher irisin levels were associated with higher odds of obesity, abdominal obesity and sarcopenic obesity; however, these associations lost significance after further adjustment for HOMA‐IR, CRP and adiponectin. Mediation analysis further indicated that HOMA‐IR significantly mediated the associations of irisin with these outcomes. Longitudinally, greater 3‐year increases in irisin were significantly associated with obesity, abdominal obesity, sarcopenia, sarcopenic obesity and low ALST at follow‐up. In contrast, adiponectin levels showed inverse associations with obesity and abdominal obesity only in the cross‐sectional analysis.

Early research on irisin showed that exercise‐induced irisin promotes brown fat‐like development in white adipose tissue [8]. Additionally, in mouse models, recombinant irisin has been shown to stimulate muscle regeneration and alleviate age‐related sarcopenia and metabolic decline [33]. However, human studies have reported conflicting findings on its relationship with obesity and sarcopenia. For example, some studies observed positive associations between irisin and BMI, body fat mass, WC and leptin [9, 10, 11], while others found negative or no associations [34, 35]. However, a meta‐analysis of 18 studies reported that individuals with overweight or obesity had higher circulating irisin levels than healthy controls [12]. Consistent with these findings, our cross‐sectional analysis revealed that individuals with obesity had higher irisin levels than those without obesity and that higher irisin levels were associated with increased odds of obesity.

With respect to sarcopenia‐related parameters, our cross‐sectional analysis found no significant associations between irisin levels and low ALST, low muscle strength or low physical performance. This finding is consistent with previous studies reporting no significant differences in circulating irisin levels between individuals with and without sarcopenia [21, 22]. However, some studies have suggested a potential role for irisin in muscle health, showing lower levels in individuals with sarcopenia and positive associations with muscle mass, handgrip strength or IGF‐1 expression [9, 19, 20]. Specifically, Chang et al. (2017), in a study of 715 Korean adults aged 18–90 years, observed that circulating irisin levels were significantly lower in both presarcopenia and sarcopenia groups compared to the nonsarcopenia group [20].

The discrepancy from our findings may be attributable to differences in participant characteristics and methodological approaches. While Chang et al. included participants across a broad age range with similar proportions of young, middle‐aged and older adults, our cross‐sectional analysis consisted primarily of older adults (median age: 74 years). In our population, elevated irisin levels may represent a compensatory response to age‐related muscle decline and metabolic stress rather than a direct reflection of muscle mass. Methodological differences may also explain these divergent results. The use of bioelectrical impedance analysis with height‐adjusted cutoffs (ALST/height^2^) in previous research may underestimate low ALST in individuals with obesity [31], whereas our method using DXA with obesity‐stratified definitions may have improved diagnostic accuracy in those with higher adiposity. Furthermore, differences in the methods of irisin measurements and individual variation in inflammation, metabolism or physical activity may have influenced the associations observed.

Additionally, in the longitudinal analysis, participants with the greatest 3‐year increases in irisin had higher odds of obesity, abdominal obesity, sarcopenia, low ALST and sarcopenic obesity at follow‐up compared to those with the least increase or with decreased irisin. Sarcopenia and obesity share common pathophysiological mechanisms, including reduced physical activity, increased oxidative stress, insulin resistance and elevated inflammatory cytokines. Their coexistence is associated with a higher risk of mortality and functional decline compared to either condition alone [5, 6]. Although obesity is generally considered protective against sarcopenia due to its association with greater absolute muscle mass [6], our findings indicate that an increase in irisin levels may be associated with a higher risk of sarcopenic obesity, potentially through simultaneous increases in abdominal adiposity and declines in muscle mass.

Although the underlying mechanisms remain unclear, evidence suggests that elevated irisin levels may represent a compensatory response to insulin resistance and metabolic dysfunction [10, 12]. This is supported by weight loss intervention studies reporting a decline in irisin levels following weight loss [9, 36], with irisin levels also shown to return to baseline levels during weight regain [36]. Additionally, similar to the concept of insulin or leptin resistance, obesity and metabolic disturbances may lead to a state of irisin resistance, wherein elevated circulating levels reflect a compensatory response to maintain glucose and metabolic homeostasis [10]. Furthermore, our finding that associations between irisin and obesity were attenuated after adjustment for HOMA‐IR and CRP suggests that these relationships are mediated through insulin resistance and inflammatory pathways. Subsequent mediation analysis revealed that HOMA‐IR, not CRP, significantly mediated the association between irisin, obesity and sarcopenic obesity. The absence of a significant direct effect suggests that irisin's influence on obesity and sarcopenic obesity is primarily mediated through insulin resistance.

The difference between cross‐sectional and longitudinal findings may be partly explained by this mediating role of insulin resistance. In cross‐sectional analysis, where exposure, mediator and outcome are measured simultaneously, the effect of irisin may be accounted for by HOMA‐IR, thereby attenuating direct associations. In contrast, longitudinal changes may better reflect sustained biological responses to progressive metabolic stress and alterations in body composition. Indeed, participants with the greatest increases in irisin also exhibited greater gains in WC and body fat mass. A single measurement may not reflect these adaptive processes, which could explain the lack of cross‐sectional associations. Our findings suggest that irisin is a dynamic biomarker reflecting metabolic alterations and may be useful for monitoring metabolic health over time.

In contrast to irisin, adiponectin levels showed inverse associations with obesity and abdominal obesity, consistent with previous research [37]. Mediation analysis further revealed that HOMA‐IR significantly mediated these associations. However, our cross‐sectional results were not entirely robust. Although the highest adiponectin tertile was inversely associated with obesity and abdominal obesity, the intermediate tertile showed positive associations, and no significant associations were observed when adiponectin was analysed as a continuous variable (per log‐unit increase) after adjustment for confounding variables. Potential explanations include attenuation of direct effects after adjustment for HOMA‐IR and the limited sample size within each tertile. Cohort‐specific factors may also have contributed, as older Asian adults present with higher body fat at lower BMI values, and healthy survivor bias may have attenuated risk in the lowest group [38].

Despite its inverse association with obesity and abdominal obesity, adiponectin was not significantly associated with sarcopenia or its related components. Although individuals in the intermediate adiponectin group showed higher odds of low ALST, this association was not observed in the highest group, and no linear trend was present (Table S1). In addition, adiponectin levels also did not differ by sarcopenia status in either sex, and no associations were observed for other sarcopenia‐related outcomes. These findings are inconsistent with a meta‐analysis of seven studies, which reported significantly higher circulating adiponectin levels among individuals with sarcopenia [24]. This paradoxical elevation has been interpreted as the ‘adiponectin paradox’ in older adults, in which elevated adiponectin levels may reflect a compensatory response to metabolic stress, including systemic inflammation, insulin resistance and muscle catabolism. Indeed, increased adiponectin levels have also been reported in conditions such as chronic kidney disease and heart failure [39, 40]. Another possible explanation is adiponectin resistance, similar to insulin or leptin resistance, where elevated circulating levels fail to exert their expected anabolic effects on muscle tissue.

Conversely, other studies have reported lower circulating adiponectin levels in individuals with sarcopenia, with lower adiponectin being independently associated with increased sarcopenia risk [16, 23]. Experimental research in aged mice has also demonstrated that adiponectin promotes muscle regeneration through the AdipoR1–AMPK signalling pathway and that inhibition of adiponectin or its receptor attenuates these exercise‐induced benefits [15]. These inconsistencies suggest that adiponectin's role in sarcopenia may differ depending on participant characteristics, inflammatory status or diagnostic criteria used to define sarcopenia. The role of adiponectin in age‐related changes in body composition, including sarcopenia, remains controversial and warrants further investigation.

To our knowledge, this is the first study to examine both cross‐sectional and longitudinal associations of irisin and adiponectin with obesity, sarcopenia and sarcopenic obesity in a population‐based cohort of older adults. However, several limitations should be considered. First, although our study included a longitudinal analysis over 3 years, causal relationships cannot be definitively established. The observed associations may be influenced by reverse causality, whereby metabolic dysfunction and obesity drive changes in irisin levels as a compensatory mechanism. Additionally, despite our extensive adjustments for known confounders including age, sex, lifestyle factors and metabolic parameters, residual confounding cannot be excluded. Future experimental studies are needed to clarify the causal pathways and determine whether these biomarkers are drivers or merely markers of metabolic dysfunction. Second, our study population included middle‐aged and primarily older Korean adults, limiting generalizability to other ethnicities and younger age groups. Asian populations typically have higher body fat percentages at lower BMI values than Western populations, which may influence the associations between biomarkers and body composition [38]. Further studies in diverse ethnic and age groups are needed to confirm these findings. Third, variations in the diagnostic criteria for sarcopenia and sarcopenic obesity across studies may have influenced comparability with previous research. Fourth, DXA measurements were available only at follow‐up, limiting our ability to determine the incidence of sarcopenia and distinguish between pre‐existing and newly developed cases. While we addressed this by adjusting for baseline biomarker levels, BMI and sarcopenia‐related parameters (muscle strength and physical performance), these adjustments may not fully capture baseline muscle mass and function. Finally, the smaller sample size of this study relative to previous large‐scale cross‐sectional studies may have limited the statistical power to demonstrate associations between irisin and adiponectin and obesity‐ and sarcopenia‐related outcomes. Future studies using larger and more ethnically diverse cohorts with serial assessments of both biomarkers and body composition are warranted to determine how changes in irisin and adiponectin are temporally associated with alterations in muscle mass and adiposity.

## 5 Conclusion

Although irisin has been proposed as a therapeutic target for metabolic disorders, our findings do not support its direct protective role in older adults. Instead, elevated circulating irisin may reflect compensatory mechanisms or resistance to metabolic dysfunction. Greater increases in irisin were associated with higher odds of obesity, abdominal obesity, sarcopenia, low ALST and sarcopenic obesity. Conversely, higher adiponectin levels had an inverse association with obesity and abdominal obesity. Further research is needed to clarify their complex interactions with fat and muscle in aging.

## Funding

This research was funded by the Hallym University Research Fund 2024 (HURF‐2024‐04).

## Conflicts of Interest

The authors declare no conflicts of interest.

## Supporting information


**Data S1:** Supplementary Information.

**Table S1:** Cross‐sectional associations of 1‐unit increase in log irisin and adiponectin with obesity‐and sarcopenia‐related outcomes (2010).
Table S2. Longitudinal associations of 1‐SD increase in irisin and adiponectin change with obesity‐and sarcopenia‐related outcomes.

**Figure S1:** Diagnostic algorithm for sarcopenia, sarcopenic obesity and severe sarcopenia.

**Figure S2:** Mediation effects of C‐reactive protein (CRP) on the associations between irisin or adiponectin and obesity (A, C) and sarcopenic obesity (B, D).
