# Supplementary material for: Cross‐Sectional and Longitudinal Associations of Irisin and Adiponectin With Obesity, Sarcopenia and Sarcopenic Obesity
Source: J Cachexia Sarcopenia Muscle. 2025 Dec 29;17(1):e70172. doi: 10.1002/jcsm.70172 (PMC12746044; doi:10.1002/jcsm.70172)
Supplement: Supplementary file 1 — Data S1: Supplementary Information. [file JCSM-17-e70172-s003.docx]

Supplementary Appendix

**Cross-Sectional and Longitudinal Associations of Irisin and Adiponectin with Obesity, Sarcopenia, and Sarcopenic Obesity**

Fig S1. Diagnostic algorithm for sarcopenia, sarcopenic obesity, and severe sarcopenia

Table S1. Cross-sectional associations of 1-unit increase in log irisin and adiponectin with obesity- and sarcopenia-related outcomes (2010).

Table S2. Longitudinal associations of 1-SD increase in irisin and adiponectin change with obesity- and sarcopenia-related outcomes

Fig S2. Mediation effects of C-reactive protein (CRP) on the associations between irisin or adiponectin and obesity (A, C) and sarcopenic obesity (B, D).
