# Supplementary material for: Cross‐Sectional and Longitudinal Associations of Irisin and Adiponectin With Obesity, Sarcopenia and Sarcopenic Obesity
Source: J Cachexia Sarcopenia Muscle. 2025 Dec 29;17(1):e70172. doi: 10.1002/jcsm.70172 (PMC12746044; doi:10.1002/jcsm.70172)
Supplement: Supplementary file 2 — Table S1: Cross‐sectional associations of 1‐unit increase in log irisin and adiponectin with obesity‐and sarcopenia‐related outcomes (2010). Table S2. Longitudinal associations of 1‐SD increase in irisin and adiponectin change with obesity‐and sarcopenia‐related outcomes. [file JCSM-17-e70172-s002.docx]

Table S1. Cross-sectional associations of 1-unit increase in log irisin and adiponectin with obesity**-** and sarcopenia-related outcomes (2010).

| **Irisin (n=357)** | | | **Adiponectin (n=360)** | | |  |
| --- | --- | --- | --- | --- | --- | --- |
|  | **1-unit increase in log irisin, OR (95% CI)** | ***P*-value** | | **1-unit increase in log adiponectin, OR (95% CI)** | ***P*-value** |  |
| **Obesity** | | | | | |  |
| Model 1 | 1.22 (0.94–1.61) | 0.138 | | 0.63 (0.41–0.96) | **0.034** |  |
| Model 2 | 1.45 (1.09–1.98) | **0.014** | | 0.53 (0.33–0.83) | **0.007** |  |
| Model 3 | 1.25 (0.92–1.72) | 0.158 | | 0.66 (0.40–1.11) | 0.116 |  |
| **Abdominal obesity** | | | | | |  |
| Model 1 | 1.12 (0.88-1.44) | 0.342 | | 0.56 (0.37-0.82) | **0.004** |  |
| Model 2 | 1.28 (0.98-1.69) | 0.072 | | 0.43 (0.27-0.67) | **<0.001** |  |
| Model 3 | 1.02 (0.76-1.37) | 0.889 | | 0.64 (0.38-1.05) | 0.081 |  |
| **Sarcopenia** | | | | | | |
| Model 1 | | 1.39 (1.06–1.85) | **0.019** | | 0.92 (0.61–1.39) | 0.697 |
| Model 2 | | 1.26 (0.95–1.71) | 0.125 | | 0.98 (0.62–1.53) | 0.923 |
| Model 3 | | 1.18 (0.88–1.61) | 0.275 | | 1.11 (0.69–1.77) | 0.675 |
| **Severe sarcopenia** | | | | | |  |
| Model 1 | 1.11 (0.76–1.65) | 0.593 | | 0.99 (0.53–1.86) | 0.984 |  |
| Model 2 | 1.07 (0.68–1.68) | 0.777 | | 0.67 (0.34–1.34) | 0.265 |  |
| Model 3 | 1.02 (0.64–1.63) | 0.939 | | 0.70 (0.34–1.41) | 0.318 |  |
| **Sarcopenic obesity** | | | | | |  |
| Model 1 | 1.54 (1.08–2.25) | **0.02** | | 0.49 (0.28–0.86) | **0.014** |  |
| Model 2 | 1.64 (1.12–2.50) | **0.015** | | 0.46 (0.24–0.84) | **0.014** |  |
| Model 3 | 1.34 (0.90–2.06) | 0.16 | | 0.55 (0.28–1.08) | 0.083 |  |
| **Low ALST** | | | | | |  |
| Model 1 | 1.45 (1.13–1.91) | **0.005** | | 0.80 (0.54–1.17) | 0.247 |  |
| Model 2 | 1.26 (0.96–1.67) | 0.107 | | 1.01 (0.66–1.55) | 0.965 |  |
| Model 3 | 1.20 (0.91–1.61) | 0.203 | | 1.08 (0.70–1.69) | 0.726 |  |
| **Low muscle strength** | | | | | | |
| Model 1 | 1.06 (0.80–1.40) | 0.693 | | 1.75 (1.12–2.77) | **0.015** |  |
| Model 2 | 1.17 (0.85–1.64) | 0.349 | | 1.21 (0.73–2.00) | 0.456 |  |
| Model 3 | 1.15 (0.82–1.63) | 0.416 | | 1.20 (0.72–2.02) | 0.486 |  |
| **Low physical performance** | | | | | | |
| Model 1 | 0.87 (0.67–1.12) | 0.283 | | 1.60 (1.06–2.43) | **0.026** |  |
| Model 2 | 0.98 (0.73–1.31) | 0.894 | | 1.09 (0.68–1.76) | 0.719 |  |
| Model 3 | 0.95 (0.70–1.29) | 0.763 | | 1.19 (0.73–1.95) | 0.498 |  |
| Abbreviations: CI, confidence interval; OR, odds ratio.  Model 1: unadjusted.  Model 2: adjusted for age, sex, current smoking, alcohol drinking, regular exercise, and comorbidity.  Model 3: Model 2 + HOMA-IR, CRP, and mutual biomarkers (adiponectin and irisin, respectively).  *P*-values <0.05 are presented in bold. | | | | | |  |

Table S2. Longitudinal associations of 1-SD increase in irisin and adiponectin change with obesity**-** and sarcopenia-related outcomes

| **Irisin (n=349)** | | | **Adiponectin (n=347)** | | |
| --- | --- | --- | --- | --- | --- |
|  | **1-SD increase in irisin change, OR (95% CI)** | ***P*-value** | | **1-SD increase in adiponectin change, OR (95% CI)** | ***P*-value** |
| **Obesity** | | | | | |
| Model 1 | 1.20 (0.94–1.55) | 0.152 | | 0.88 (0.70–1.11) | 0.292 |
| Model 2 | 1.21 (0.93–1.59) | 0.173 | | 0.79 (0.59–1.05) | 0.11 |
| Model 3 | 1.31 (0.99–1.76) | 0.066 | | 0.81 (0.60–1.08) | 0.156 |
| **Abdominal obesity** | | | | | |
| Model 1 | 1.22 (0.98–1.55) | 0.082 | | 0.92 (0.74–1.13) | 0.417 |
| Model 2 | 1.19 (0.89–1.60) | 0.256 | | 0.67 (0.49–0.91) | **0.011** |
| Model 3 | 1.42 (1.02–2.02) | **0.045** | | 0.68 (0.48–0.95) | **0.027** |
| **Sarcopenia** | | | | | |
| Model 1 | 0.97 (0.78–1.23) | 0.825 | | 1.08 (0.86–1.35) | 0.518 |
| Model 2 | 1.23 (0.96–1.59) | 0.107 | | 0.96 (0.73–1.25) | 0.754 |
| Model 3 | 1.26 (0.97–1.65) | 0.083 | | 0.97 (0.73–1.28) | 0.824 |
| **Severe sarcopenia** | | | | | |
| Model 1 | 1.01 (0.73–1.46) | 0.944 | | 0.88 (0.63–1.24) | 0.47 |
| Model 2 | 1.42 (0.88–2.27) | 0.14 | | 0.61 (0.37–0.97) | **0.045** |
| Model 3 | 1.46 (0.88–2.44) | 0.138 | | 0.55 (0.31–0.93) | **0.035** |
| **Sarcopenic obesity** | | | | | |
| Model 1 | 1.17 (0.86–1.64) | 0.341 | | 0.91 (0.68–1.23) | 0.557 |
| Model 2 | 1.37 (0.98–1.94) | 0.071 | | 0.67 (0.43–1.00) | 0.064 |
| Model 3 | 1.46 (1.03–2.11) | **0.039** | | 0.70 (0.43–1.07) | 0.118 |
| **Low ALST** | | | | | |
| Model 1 | 1.06 (0.85–1.32) | 0.614 | | 1.00 (0.81–1.24) | 0.984 |
| Model 2 | 1.24 (0.97–1.59) | 0.085 | | 0.90 (0.70–1.15) | 0.401 |
| Model 3 | 1.26 (0.98–1.63) | 0.075 | | 0.91 (0.70–1.17) | 0.463 |
| **Low muscle strength** | | | | | |
| Model 1 | 0.91 (0.72–1.16) | 0.421 | | 0.92 (0.72–1.18) | 0.515 |
| Model 2 | 1.23 (0.87–1.76) | 0.237 | | 0.93 (0.69–1.25) | 0.65 |
| Model 3 | 1.18 (0.81–1.72) | 0.393 | | 0.81 (0.57–1.13) | 0.226 |
| **Low physical performance** | | | | | |
| Model 1 | 0.87 (0.68–1.09) | 0.24 | | 1.19 (0.95–1.49) | 0.138 |
| Model 2 | 1.04 (0.79–1.38) | 0.786 | | 1.12 (0.84–1.52) | 0.443 |
| Model 3 | 1.10 (0.82–1.50) | 0.534 | | 1.10 (0.80–1.52) | 0.573 |
| Abbreviations: CI, confidence interval; OR, odds ratio.  Model 1: Unadjusted.  Model 2: Adjusted for baseline irisin or adiponectin levels, BMI (WC for abdominal obesity), and sarcopenia-related variables. Specifically, sarcopenia, sarcopenic obesity, and low ALST were adjusted for baseline handgrip strength, chair stand test, and gait speed. Low muscle strength was adjusted for baseline handgrip strength only, and low physical performance for baseline chair stand test and gait speed.  Model 3: Model 2 adjustments plus age, sex, current smoking, alcohol drinking, regular exercise, comorbidity, HOMA-IR, CRP, and mutual biomarkers (adiponectin and irisin, respectively).  Values exceeding ±3 SD in irisin or adiponectin change were excluded (n = 2 for irisin; n = 4 for adiponectin).  *P*-values <0.05 are presented in bold. | | | | | |
