# Supplementary figures and images for: Cross‐Sectional and Longitudinal Associations of Irisin and Adiponectin With Obesity, Sarcopenia and Sarcopenic Obesity
Source: J Cachexia Sarcopenia Muscle. 2025 Dec 29;17(1):e70172. doi: 10.1002/jcsm.70172 (PMC12746044; doi:10.1002/jcsm.70172)

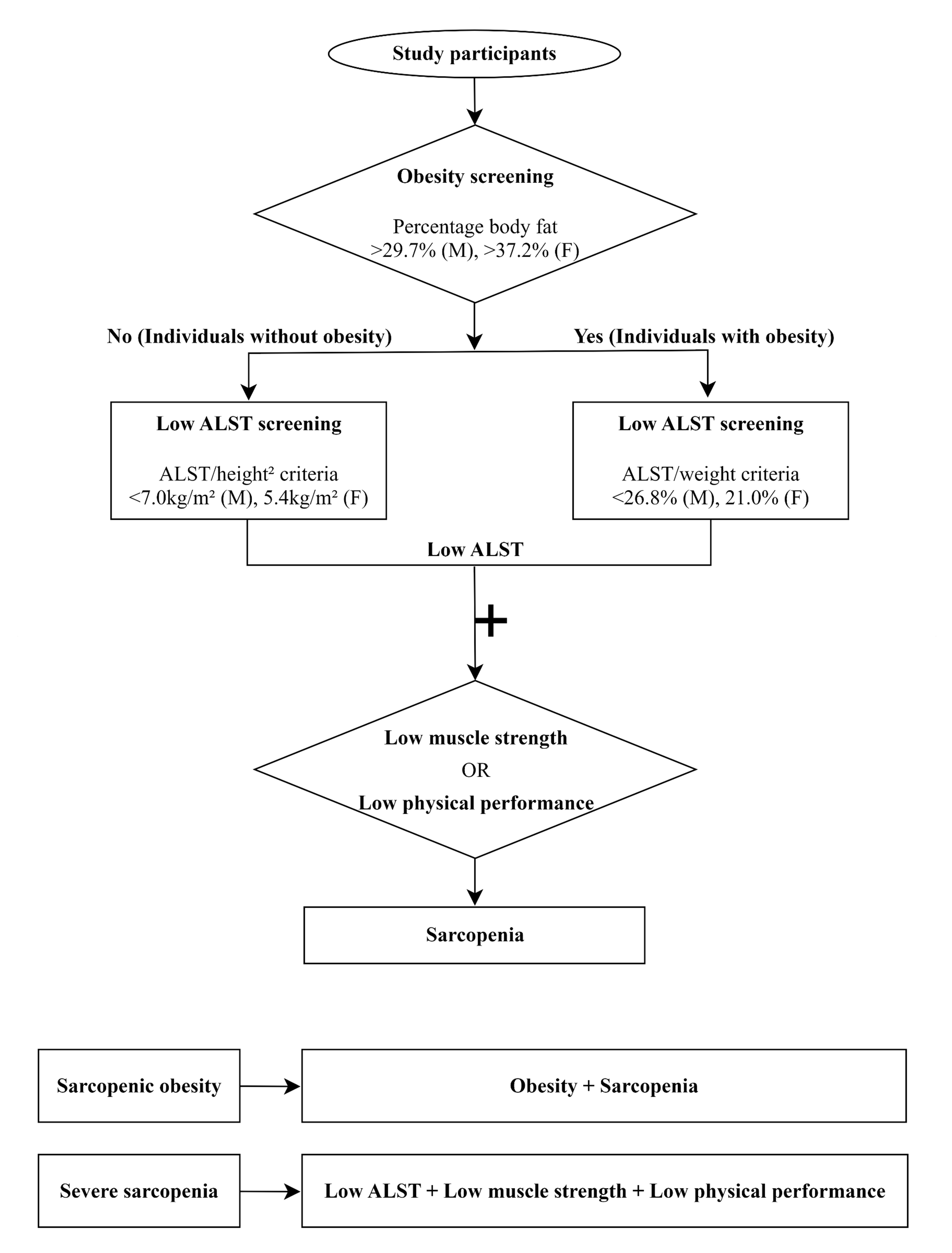

Supplement: Supplementary file 3 — Figure S1: Diagnostic algorithm for sarcopenia, sarcopenic obesity and severe sarcopenia. [file JCSM-17-e70172-s001.tif]

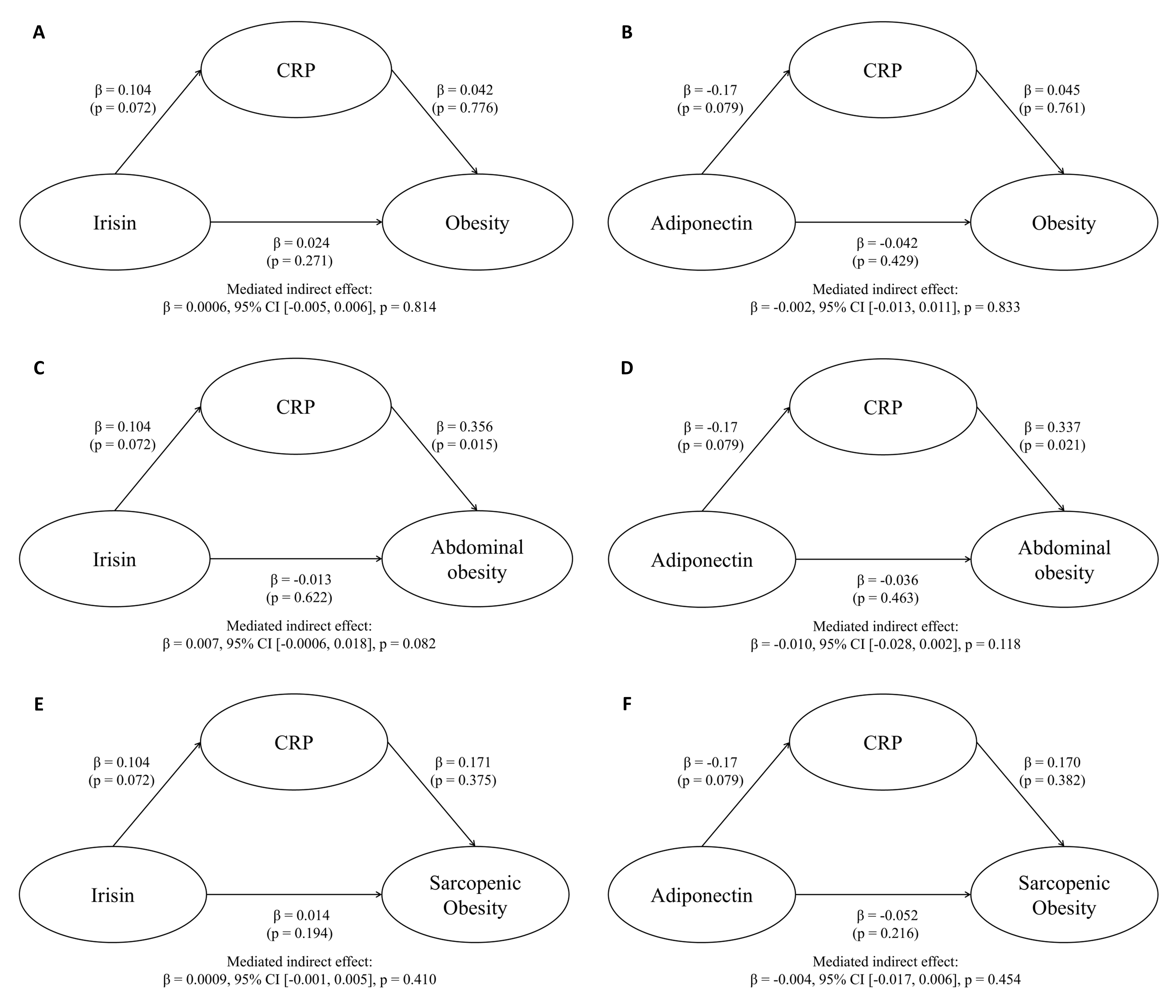

Supplement: Supplementary file 4 — Figure S2: Mediation effects of C‐reactive protein (CRP) on the associations between irisin or adiponectin and obesity (A, C) and sarcopenic obesity (B, D). [file JCSM-17-e70172-s004.tif]
